# Effect of a New Skin‐Lightening Cosmetic Containing Cordyceps Extract in the Treatment of Melasma: A Clinical Trial

**DOI:** 10.1111/jocd.70329

**Published:** 2025-07-01

**Authors:** Sihao Shen, Huiyi Yao, Yuan Zhu, Wenzhong Xiang

**Affiliations:** ^1^ Department of Medical Cosmetology Zhejiang Chinese Medicine and Western Medicine Integrated Hospital Hangzhou Zhejiang China; ^2^ Department of Dermatology Hangzhou Third People's Hospital, Zhejiang Chinese Medical University Hangzhou Zhejiang China; ^3^ Department of Dermatology Hangzhou Third People's Hospital, Hangzhou Third People's Hospital Affiliated to Zhejiang Chinese Medical University Hangzhou Zhejiang China

**Keywords:** cordyceps essence, melasma, tranexamic acid

## Abstract

**Background:**

Cordyceps is a valuable Chinese herbal medicine known for its various components with antioxidant properties, which may theoretically improve melasma. This study aimed to evaluate the efficacy of a new skin‐lightening cosmetic containing Cordyceps extract (referred to as Cordyceps essence) in treating female patients with melasma.

**Methods:**

Sixty‐two women with melasma were enrolled and randomly assigned to two groups for 12 weeks of treatment. Group A received oral tranexamic acid (TXA) combined with topical hydroquinone cream, while Group B received oral TXA combined with topical Cordyceps essence. Changes in the Melasma Area and Severity Index (MASI), melanin index (MI), and erythema index (EI) were monitored and assessed before and after treatment. Patient‐reported satisfaction and adverse events were also recorded. Additionally, a metabolomic analysis was conducted on 15 randomly selected patients from Group B.

**Results:**

After 12 weeks of treatment, intra‐group comparisons revealed that MASI scores, MI, and EI significantly decreased in both Group A and B compared to baseline (*p* < 0.05). However, inter‐group comparisons showed no statistical differences in MASI scores, MI, or EI between the two groups after treatment (*p* > 0.05). Adverse reactions occurred in 4 people (13.8%) in Group A and 1 person (3.3%) in Group B. Patient satisfaction with treatment was similar in both groups. The metabolomic analysis identified significant differences in 29 metabolites and 15 metabolic pathways after treatment (*p* < 0.05).

**Conclusions:**

Our study demonstrated that both oral TXA combined with hydroquinone cream and oral TXA combined with Cordyceps essence significantly improved melasma in women. However, the incidence of adverse reactions was lower with topical Cordyceps essence than that with hydroquinone cream. Cordyceps essence appeared to be a promising alternative for patients intolerant to hydroquinone cream. Metabolomic analysis revealed that modulation of melanogenesis‐related metabolites, enhanced antioxidant activity, and improved skin barrier function collectively contributed to the clinical improvement in melasma severity. The improvement of melasma with oral TXA and topical Cordyceps essence may be closely linked to changes in endogenous differential metabolites in the skin and the regulation of amino acid metabolic pathways.

## 1 Introduction

Melasma, a skin condition characterized by symmetrical, brown, irregular patches on sun‐exposed areas, particularly the face, is especially common in Asian women in their 30s and 40s [1]. It is an acquired condition, and while the exact pathogenesis is not fully understood, factors such as prolonged ultraviolet (UV) exposure, female hormonal stimulation, and genetic predisposition are believed to contribute to its development [2]. Although melasma is not life‐threatening, it can lead to significant psychological effects, such as anxiety and depression, which negatively impact patients' quality of life [3].

Topical treatment remains the first‐line approach for managing melasma [4]. Hydroquinone is a widely used treatment due to its well‐established skin‐lightening properties; however, its safety remains controversial. Adverse reactions, such as contact dermatitis, exogenous aging, and depigmentation, have diminished its acceptance among melasma patients [5]. Therefore, there is a need for safer topical treatments.

In recent years, natural ingredients have garnered significant attention in the field of skin‐lightening cosmetics, being considered greener and safer alternatives. In this study, we conducted a randomized controlled trial (RCT) involving 62 women with melasma to evaluate the efficacy and safety of a novel skin‐lightening cosmetic containing Cordyceps extract (referred to as Cordyceps essence) for melasma treatment. This essence includes Cordyceps extract, arbutin, nonapeptide‐1, and glabridin, targeting multiple aspects of melasma's pathogenesis, which theoretically can improve the condition.

## 2 Methods

### 2.1 Study Design

The RCT was conducted at the Dermatology Department of Hangzhou Third People's Hospital from June 2023 to January 2024. Detailed explanations of the trial requirements were provided to all recruited patients, including information about potential adverse reactions and risks.

### 2.2 Study Subjects

Sixty‐two women with melasma were enrolled and randomly assigned to two groups for 12 weeks of treatment. Inclusion criteria included (1) female patients diagnosed with melasma by a physician and (2) patients agreed not to undergo any other treatment for melasma during the study period. Exclusion criteria included (1) pregnant or breastfeeding patients and those with autoimmune diseases (such as malignant tumors, lupus erythematosus, or dermatomyositis); (2) patients with other skin diseases (such as active acne or traumatic facial lesions); and (3) patients who received melasma treatment within 3 months before the study.

### 2.3 Test Product: Cordyceps Essence

This Cordyceps essence, manufactured by Sinclair Aesthetics (Shanghai) Medical Technology Co. Ltd., contains Cordyceps extract (0.2%), arbutin (0.5%), nonapeptide‐1 (3%), and glabridin (3%). Both this product and the hydroquinone cream used in the control group are certified by the China Food and Drug Administration (CFDA) (G2023007685; H20040088).

### 2.4 Interventions

The 62 women with melasma were randomly divided into two groups. Randomization was performed using a random number table with equal allocation. Group A consisted of 31 patients treated with oral TXA combined with topical hydroquinone cream. Group B included 31 patients treated with oral TXA combined with topical Cordyceps essence. Before using the essence for the first time, all participants were asked to apply a small amount of the essence behind the ear, and if there were no adverse reactions such as redness and itching within 48 h, then apply it to the facial skin. All participants were informed of necessary precautions, including sun protection, a light diet, and adequate rest.

### 2.5 Outcomes and Evaluation Methods

This RCT lasted for 12 weeks, with evaluations at baseline and at 4, 8, and 12 weeks post‐treatment. At baseline and 12 weeks after treatment, the same physician used VISIA to photograph the patients after cleansing their faces. Melasma Area and Severity Index (MASI) assessments were performed on VISIA images by two experienced physicians who were blinded to the group assignments. In addition, a Mexameter non‐invasive skin detector was utilized to measure melanin index (MI) and erythema index (EI). The same physician placed the Mexameter probe vertically on the patient's affected area, applying even pressure to ensure contact with the skin. MI and EI values were measured three times, and the average value was calculated. Changes in MASI scores, MI, and EI were compared after treatment.

At 12 weeks post‐treatment, a questionnaire was administered to evaluate patient satisfaction based on subjective assessments of improvement. Specific criteria were as follows: improvement < 25% = not satisfied; improvement of 25%–49% = partially satisfied; improvement of 50%–75% = satisfied; improvement of > 75% = very satisfied. Adverse reactions, including pruritus, pain, edema, desquamation, pigmentation, and hypopigmentation, were also monitored following treatment.

### 2.6 Metabolomic Study Methods

#### 2.6.1 Sample Collection

At baseline and 12 weeks after treatment, 15 patients from Group B were randomly selected for sample collection. After resting in a quiet environment for 15 min, D‐squame tape was used to collect skin cuticle samples from the melasma lesions. The tape was applied to the lesion area and gently pressed to ensure complete contact with the skin. After waiting for 3 min, the tape was removed at a constant speed and placed into a 2.0 mL cryogenic vial, then immediately stored in a −80°C medical cryogenic box for metabolomic analysis.

#### 2.6.2 Samples Extraction

The samples were slowly thawed at 4°C, and an appropriate amount was placed into a pre‐cooled mixture of methanol, acetonitrile, and water (in a ratio of 2:2:1). The mixture was thoroughly vortexed, then sonicated at low temperature for 30 min, and left at −20°C for 10 min. After centrifugation at 4°C for 20 min at 14 000×*g*, the supernatant was collected and dried using vacuum drying technology. Before mass spectrometry, the supernatant should be re‐dissolved in 100 μL of acetonitrile solution (1:1 ratio), vortexed, and centrifuged again at 14 000×*g* at 4°C for 15 min to prepare for analysis.

#### 2.6.3 Chromatographic Conditions

Samples were separated using an Agilent 1290 Infinity LC ultra‐high performance liquid chromatography (UHPLC) system with HILIC and C18 columns. Throughout the analysis, samples were kept in an automatic injector at 4°C. In order to minimize the impact of signal fluctuations, the samples were analyzed in random order, and quality control (QC) samples were added to the queue to monitor the system's stability and ensure data reliability.

#### 2.6.4 Mass Spectrometry Conditions

Mass spectrometry was performed using an AB 6500 + QTRAP mass spectrometer. ESI source conditions were as follows: Source temperature at 580°C, Ion Source Gas 1 at 45, Ion Source Gas 2 at 60, Curtain Gas at 35, and Ion Spray Voltage at +4500 V or−4500 V in positive or negative modes. MRM mode was used for monitoring.

#### 2.6.5 Data Analysis

Analyst software was used to extract the peak from the original MRM data, and the ratio of each substance's peak area to the internal standard peak area was calculated. Concentrations were determined using a standard curve and corrected based on the actual sample weight. The extracted data were evaluated for quality before further analysis. Data analysis included univariate and multivariate statistical analyses, identification of significantly different metabolites, cluster analyses, correlation assessments among differentiated metabolites, and metabolic pathway analysis using the Kyoto Encyclopedia of Genes and Genomes (KEGG).

### 2.7 Statistical Analysis

Data from this clinical trial were analyzed using SPSS 25.0, with GraphPad Prism 9 used for graphical representation. A *p*‐value of < 0.05 was considered statistically significant.

Principal component analysis (PCA) and orthogonal partial least squares discrimination analysis (OPLS‐DA) were performed to assess metabolite changes after treatment. PCA, an unsupervised data analysis method, was used to generally reflect the distribution patterns and differences in metabolites pre‐nd post‐treatment. OPLS‐DA, a supervised method, was applied to filter out noise unrelated to classification information, thereby enhancing the model's explanatory power, improving prediction accuracy, and maximizing the difference in metabolites after treatment. Variables were selected based on a variable influence on projection (VIP) value > 1 and the *p‐*value < 0.05.

## 3 Results

### 3.1 Case Collection and Completion Status

**FIGURE 1:**
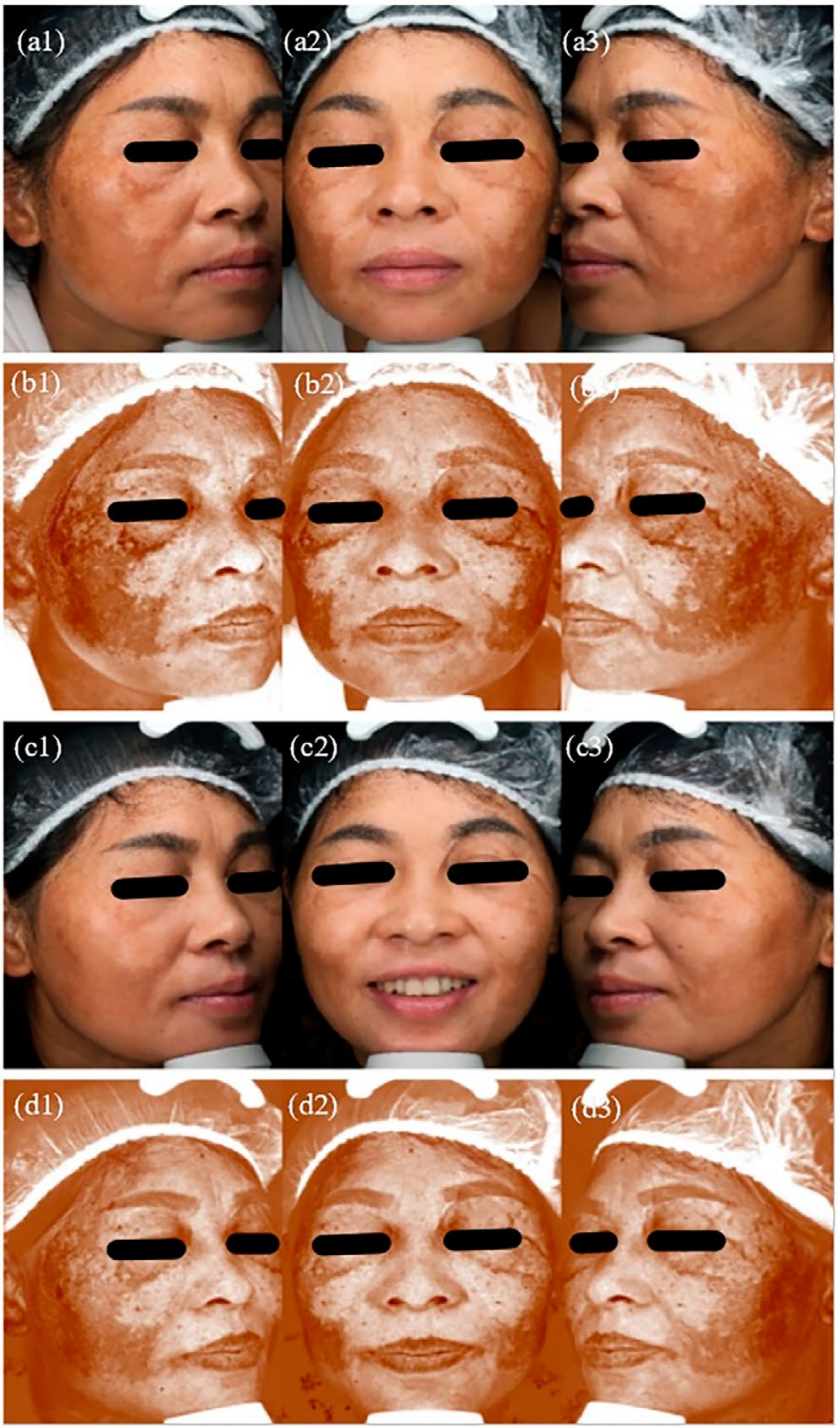
Photos and VISIA images of group A before and after treatment. (a1) Baseline photo of the right face. (a2) Baseline photo of the front face. (a3) Baseline photo of the left face. (b1) Baseline VISIA image of the right face. (b2) Baseline VISIA image of the front face. (b3) Baseline VISIA image of the left face. (c1) Post‐treatment photo of the right face. (c2) Post‐treatment photo of the front face. (c3) Post‐treatment photo of the left face. (d1) Post‐treatment VISIA image of the right face. (d2) Post‐treatment VISIA image of the front face; (d3) Post‐treatment VISIA image of the left face.

**FIGURE 2:**
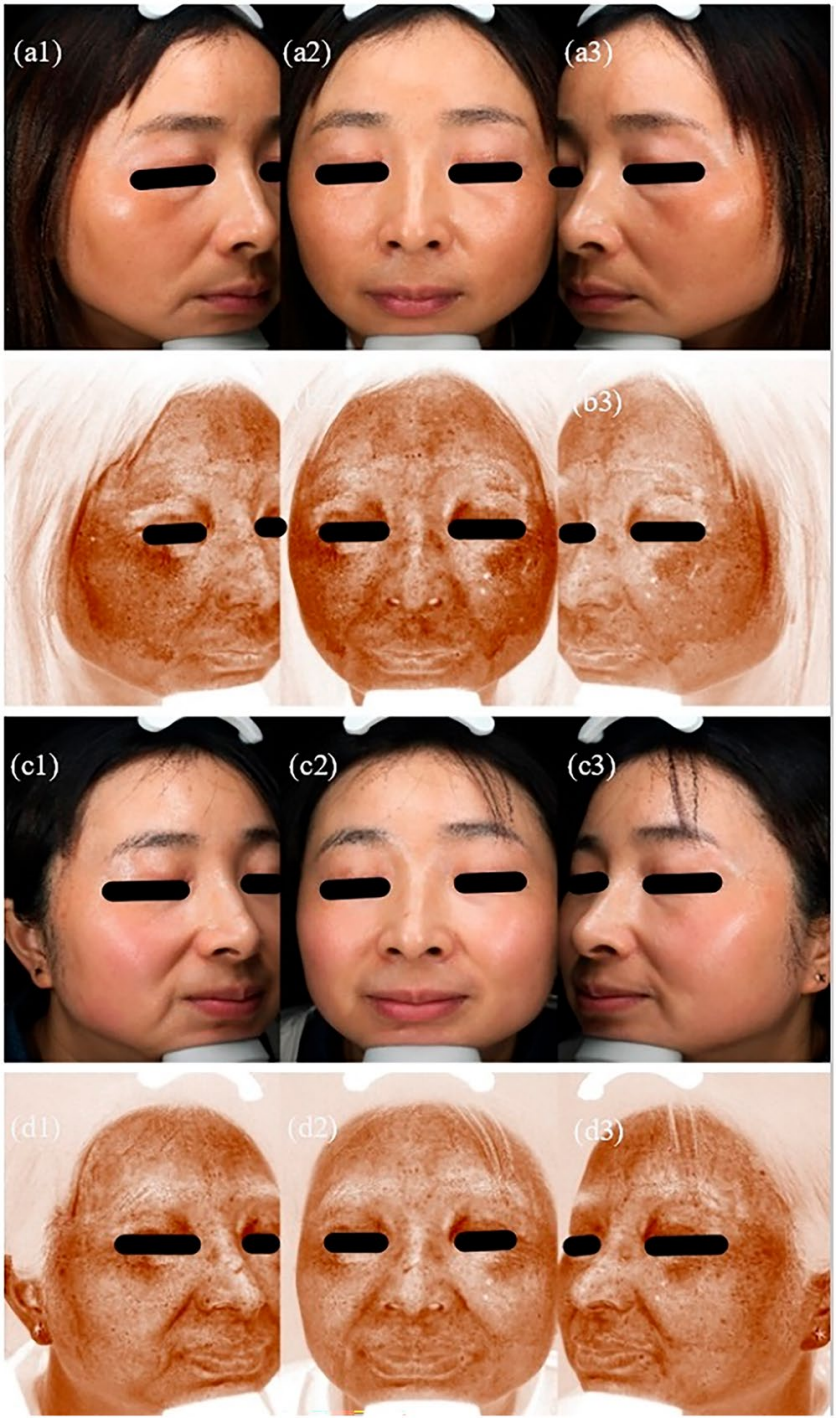
Photos and VISIA images of group B before and after treatment. (a1) Baseline photo of the right face. (a2) Baseline photo of the front face. (a3) Baseline photo of the left face. (b1) Baseline VISIA image of the right face. (b2) Baseline VISIA image of the front face. (b3) Baseline VISIA image of the left face. (c1) Post‐treatment photo of the right face. (c2) Post‐treatment photo of the front face. (c3) Post‐treatment photo of the left face. (d1) Post‐treatment VISIA image of the right face. (d2) Post‐treatment VISIA image of the front face. (d3) Post‐treatment VISIA image of the left face.

A total of 62 patients were enrolled in this clinical study, with 31 in Group A and 31 in Group B. During the study, 2 patients from Group A were excluded due to loss of follow‐up, and 1 patient from Group B was excluded for being unable to complete treatment. Finally, 29 patients in Group A and 30 patients in Group B completed the clinical trial. Pre‐ and post‐treatment photographs of Group A and Group B patients were shown in Figures 1 and 2, respectively.

### 3.2 Patient Baseline Data

The age data for both two groups followed the normal distribution, so a two‐sample independent *t*‐test was used to compare age differences. The results were *t* = −0.449, *p* = 0.655, indicating no statistically significant difference (*p* > 0.05), as shown in Table 1. The disease duration data did not follow a normal distribution, so the rank‐sum test for two independent samples was used to compare differences in disease duration. The results were *Z* = −0.390 and *p* = 0.697, also indicating no statistically significant difference (*p* > 0.05), as shown in Table 2.

**TABLE 1:** Age comparison between the two groups $\left(\bar{x}\;\pm \;s\right)$.

Group	Case (*n*)	Age (Y)	*t*	*p*
Group A	29	42.76 ± 7.26	−0.449	0.655
Group B	30	43.50 ± 5.30		

**TABLE 2:** Disease duration comparison between the two groups (*P*_25_, *P*_75_).

Group	Case (*n*)	Disease duration (Y)	*Z*	*p*
Group A	29	4.00 (3.00, 10.00)	−0.390	0.697
Group B	30	5.00 (3.00, 7.25)		

### 3.3 Comparison of MASI Scores Before and After Treatment

**FIGURE 3:**
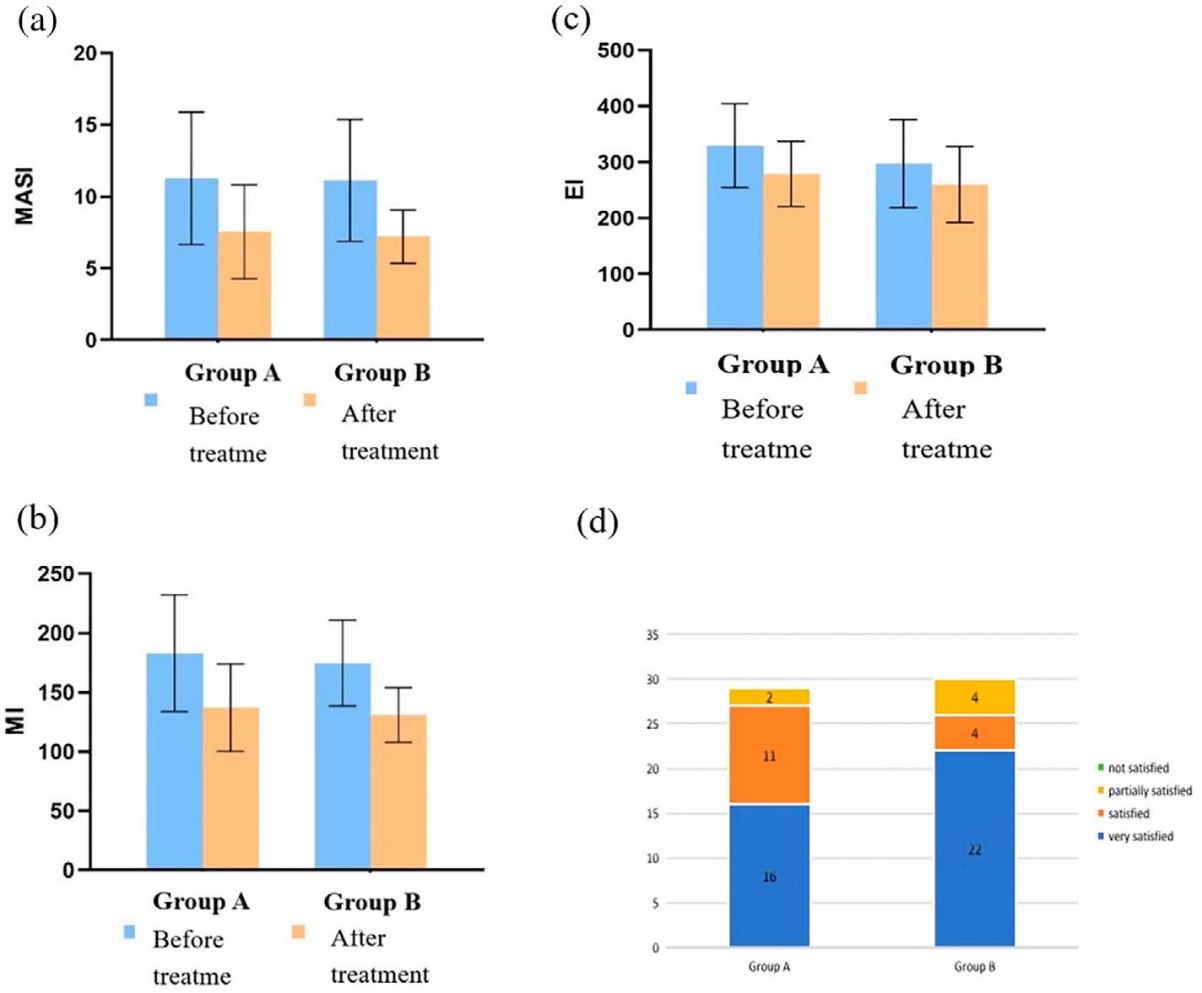
Therapeutic outcomes after 12 weeks. Changes in (a) MASI score, (b) MI, (c) EI, and (d) patient satisfaction with treatment.

The comparison of MASI scores before and after treatment is presented in Table 3 and Figure 3a.

**TABLE 3:** Comparison of MASI scores before and after treatment.

Group	MASI scores before treatment	MASI scores after treatment	Mean percentage reduction	*t* ^a^	*p* ^a^
Group A	11.28 ± 4.61	7.55 ± 3.28	33.07%	7.277	< 0.001
Group B	11.13 ± 4.25	7.20 ± 1.86	35.31%	5.651	< 0.001
*t* ^b^	0.126	0.499			
*p* ^b^ ^s^	0.900	0.620			

^a^ Paired sample *t‐*test was used for intra‐group comparison, and *p* < 0.001 indicated that there was a significant difference before and after treatment.

^b^ Two independent samples *t*/*t*‐test was used for inter‐group comparison, and *p* > 0.05 indicated that there was no significant difference between the two groups.

### 3.4 Comparison of MI Before and After Treatment

The comparison of MI before and after treatment is shown in Table 4 and Figure 3b.

**TABLE 4:** Comparison of MI before and after treatment.

Group	MI before treatment	MI after treatment	Mean percentage reduction	*t* ^a^	*p* ^a^
Group A	182.83 ± 49.07	137.10 ± 36.89	25.01%	6.867	< 0.001
Group B	174.63 ± 36.16	130.93 ± 22.90	25.02%	8.167	< 0.001
*t* ^b^	0.732	0.769			
*p* ^b^	0.467	0.446			

^a^ Paired sample *t*‐test was used for intra‐group comparison, and *p* < 0.001 indicated that there was a significant difference before and after treatment.

^b^ Two independent samples *t*/*t*‐test was used for inter‐group comparison, and *p* > 0.05 indicated that there was no significant difference between the two groups.

### 3.5 Comparison of EI Before and After Treatment

The comparison of EI before and after treatment is shown in Table 5 and Figure 3c.

**TABLE 5:** Comparison of EI before and after treatment.

Group	EI before treatment	EI after treatment	Mean percentage reduction	*t* ^a^	*p* ^a^
Group A	329.38 ± 75.12	279.14 ± 58.18	15.25%	4.911	< 0.001
Group B	297.27 ± 78.68	260.20 ± 67.89	12.47%	5.013	< 0.001
*t* ^b^	1.602	1.149			
*p* ^b^	0.115	0.255			

^a^ Paired sample *t*‐test was used for intra‐group comparison, and *p* < 0.001 indicated that there was a significant difference before and after treatment.

^b^ Two independent samples *t*/*t*‐test was used for inter‐group comparison, and *p* > 0.05 indicated that there was no significant difference between the two groups.

### 3.6 Satisfaction Evaluation

As shown in Figure 3d, self‐reported satisfaction in Group A was as follows: 0 patients were not satisfied, 2 patients (6.9%) were partially satisfied, 11 patients (37.9%) were satisfied, and 16 patients (55.2%) were very satisfied, resulting in an overall satisfaction rate of 93.1%. In Group B, 0 patients were dissatisfied, 4 patients (13.3%) were partially satisfied, 4 patients (13.3%) were satisfied, and 22 patients (73.3%) were very satisfied, leading to an overall satisfaction rate of 86.7%. A chi‐square test showed no significant difference in satisfaction between the two groups (*χ* ^2^ = 0.669, *p* = 0.413 > 0.05).

### 3.7 Adverse Reactions

In Group A, 4 patients (13.8%) experienced mild facial erythema and slight tingling, which improved within 1–2 days after discontinuing hydroquinone cream. In Group B, only 1 patient (3.3%) developed facial pruritus, which resolved after 1 day of using a topical moisturizing cream. Adverse reactions were lower in Group B than in Group A. No serious adverse reactions, such as hyperpigmentation or depigmentation, occurred during the treatment.

### 3.8 Metabolomic Study Before and After Treatment With Oral TXA and Topical Cordyceps Essence

**FIGURE 4:**
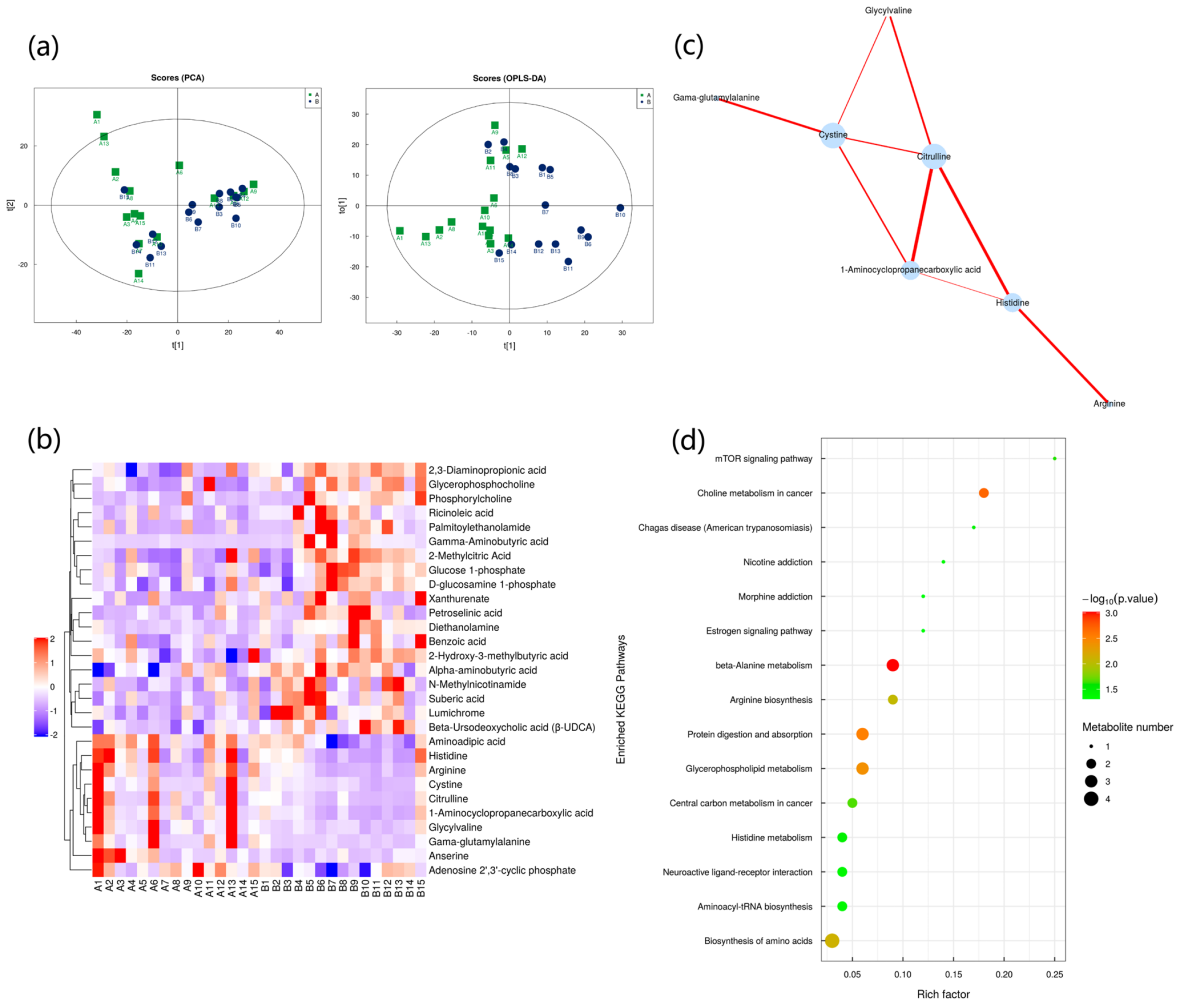
Multivariate data analysis of metabolomic study before and after melasma treatment. (a) Scoring plot of PCA and OPLS‐DA depicting differential metabolites after melasma treatment. (b) Heat map of 29 metabolites with significant differences after melasma treatment. (c) Network map of 29 metabolites with significant differences after melasma treatment. (d) Bubble map of 15 metabolic pathways with significant differences after melasma treatment.

In positive and negative ion switching modes (Figure 4a), PCA demonstrated a good clustering of skin samples before and after treatment. Although there was some overlap, a significant separation was observed along the first principal component, indicating that the skin tissues of melasma patients exhibited significant metabolic changes after treatment. The OPLS‐DA model further filtered out unrelated noise, clearly separated the two sample groups, and confirmed notable differences in skin metabolites pre and post‐treatment.

A total of 404 metabolites were identified, with 29 metabolites showing statistically significant changes (*p* < 0.05), as detailed in Table 6 and Figure 4b. Among these, 10 metabolites increased in concentration, while 19 decreased. Correlation analysis revealed that cystine, citrulline, 1‐aminocyclopropanecarboxylic acid, and histidine played key roles in this process (Figure 4c). KEGG pathway analysis showed that 15 of the 49 metabolic pathways involved displayed significant differences after treatment (*p* < 0.05), as shown in Figure 4d.

**TABLE 6:** The difference in metabolites after treatment.

Number	Differential metabolites	*p*	Content changes
1	1‐aminocyclopropanecarboxylic acid	0.024	↑
2	2,3‐diaminopropionic acid	0.006	↓
3	2‐hydroxy‐3‐methylbutyric acid	0.034	↓
4	2‐methylcitric acid	0.035	↓
5	Adenosine 2′,3′‐cyclic phosphate	0.010	↑
6	Alpha‐aminobutyric acid	0.008	↓
7	Aminoadipic acid	0.003	↑
8	Anserine	0.032	↑
9	Arginine	0.027	↑
10	Benzoic acid	0.035	↓
11	Beta‐ursodeoxycholic acid	0.002	↓
12	Citrulline	0.037	↑
13	Cystine	0.048	↑
14	d‐glucosamine 1‐phosphate	0.033	↓
15	Diethanolamine	0.016	↓
16	Gama‐glutamyl alanine	0.029	↑
17	Gamma‐aminobutyric acid	0.027	↓
18	Glucose 1‐phosphate	0.042	↓
19	Glycerophosphocholine	0.018	↓
20	Glycylvaline	0.042	↑
21	Histidine	0.044	↑
22	Lumichrome	0.018	↓
23	n‐methylnicotinamide	0.024	↓
24	Palmitoylethanolamide	0.031	↓
25	Petroselinic acid	0.018	↓
26	Phosphorylcholine	0.032	↓
27	Ricinoleic acid	0.017	↓
28	Suberic acid	0.028	↓
29	Xanthurenate	0.040	↓

*Note:* ↑: increase; ↓: decrease.

## 4 Discussion

Melasma is an acquired hyperpigmentation disorder primarily affecting the face, commonly seen in individuals with Fitzpatrick skin types III–V. The condition is chronic, often lasting 10–20 years, and recurs frequently without strict sun avoidance. Factors such as genetic predisposition, sun exposure, and female hormones are known contributors [6]. Recent research has uncovered additional factors that may play a role in the development of melasma. Clinical studies have revealed elevated levels of oxidative stress markers and enzymes in melasma patients. Vikrant et al. found that malondialdehyde (MDA) and glutathione levels were significantly higher in patients with melasma than in controls, indicating increased oxidative stress [7]. Additionally, a study on Chinese medicine combined with He–Ne laser treatment showed improved antioxidant capacity and reduced nitric oxide (NO) levels in patients, leading to significant melasma improvement. This suggests an imbalance between oxidative stress and antioxidant defenses is linked to the condition [8]. These insights provide new opportunities for developing skin care products with freckle‐lightening properties.

Topical treatment remains the first‐line therapy for melasma, including topical drugs, chemical peels, and laser treatments, though results vary. Hydroquinone, a commonly used prescription drug, is considered the gold standard for melasma treatment. However, its adverse reactions, such as skin irritation, allergic reactions, and, in severe cases, permanent depigmentation and exogenous aging, have raised concerns among dermatologists [9, 10]. Chemical peels can serve as adjunctive treatments but are limited in use, especially for patients with active melasma or compromised skin barriers [11]. Laser therapy can be effective, but post‐treatment hyperpigmentation, particularly in individuals with darker skin, is a common side effect [12]. As a result, current topical treatments for melasma remain less than satisfactory. In recent years, natural ingredients have gained widespread attention in the field of skin‐lightening cosmetics, with increasing demand from patients for products containing natural compounds.

Cordyceps, a valuable traditional Chinese medicinal ingredient, is known for its high nutritional value and biological activity. Derived from fungi of the genus Cordyceps, it is widely used as a tonic for boosting health, and its biological roles are well documented [13]. Cordyceps, especially from Cordyceps cicadae, has notable antioxidant and anti‐aging properties. It can increase catalase (CAT) and glutathione peroxidase (GSH‐Px) activities, inhibit MDA production, and upregulate antioxidant‐related genes [14, 15]. More importantly, Cordyceps extracts ergothioneine, the core ingredient of the product studied. Ergothioneine has been proven to be a potent antioxidant, capable of directly regulating reactive oxygen species (ROS) levels in cells and mitochondria and activating SIRTs and Nrf2 pathways to enhance antioxidant defenses and reduce oxidative stress [16, 17]. In the nucleus, ergothioneine rapidly chelates metal ions such as iron, copper, and zinc without producing harmful free radicals via the Fenton reaction, thereby preventing cellular and DNA damage [18]. Due to its safety profile, antioxidant properties, targeted anti‐inflammatory abilities, anti‐aging effects, non‐competitive whitening capabilities, and excellent stability, ergothionein has become a popular ingredient in skincare. In addition, Cordyceps contains extracellular polysaccharides, ergosterol, and other components that also possess antioxidant and anti‐inflammatory properties [19, 20].

The skin‐lightening cosmetic also includes arbutin, nonapeptide‐1, and glabridin. Compared to hydroquinone, arbutin is less irritating, safer, and offers stronger tyrosinase (TYR) inhibition, lower cytotoxicity, and antioxidant function, making it a safe and effective skin‐whitening ingredient [21]. Nonapeptide‐1, a synthetic melanocyte‐stimulating hormone antagonist, inhibits melanin production and is widely used in skincare and dermatology [22]. Glabridin, an isoflavone derived from licorice root, possesses anti‐inflammatory properties and inhibits TYR activity, reducing melanin production [23]. Together, these ingredients suggest that the cosmetic product has promising potential for the adjunctive treatment of melasma.

Oral TXA combined with topical hydroquinone cream is a common treatment for melasma, with previous clinical trials showing that the combination is more effective than hydroquinone alone. In a study by Shihab et al., 50 patients with melasma were randomly assigned to receive either oral TXA with topical hydroquinone or hydroquinone alone. After 12 weeks, the MASI scores in the combination group decreased by 55%, compared to only a 10.9% reduction in the hydroquinone‐alone group, with statistically significant differences [24]. Similarly, Lajevardi et al. reported that combining topical hydroquinone and oral TXA improved melasma treatment efficacy [25]. Therefore, combination therapy is considered the preferred treatment approach for melasma.

This study compared the changes in MASI scores, MI, and EI in the treatment of melasma using two regimens: oral TXA combined with topical hydroquinone cream and oral TXA combined with topical Cordyceps essence. The inter‐group comparison revealed no significant differences in MASI scores, MI, and EI between the two groups before treatment (*p* > 0.05). After 12 weeks of treatment, intra‐group comparison showed a significant reduction in MASI scores, MI, and EI in both groups, with statistical significance (*p* < 0.05). This demonstrated that both treatment regimens were effective in significantly improving melasma. However, after 12 weeks, the inter‐group comparison still showed no significant difference in MASI scores, MI, and EI between the two groups (*p* > 0.05), making it inconclusive which regimen was more effective.

A chi‐square test showed no significant difference in patient satisfaction between the two groups (*p* > 0.05). While the proportion of satisfied patients in Group A (93.1%) was slightly higher than in Group B (86.7%), the proportion of very satisfied patients in Group B (73.3%) exceeded that of Group A (55.2%). Overall, patient feedback on both treatments was positive, with no dissatisfaction expressed, indicating that the patients recognized the efficacy.

In terms of adverse reactions, 4 patients (13.8%) in Group A developed mild facial erythema with a tingling sensation, while 1 patient (3.3%) in Group B experienced facial pruritus, which resolved with treatment. No other adverse reactions, such as pigmentation or depigmentation, were observed. The lower incidence of adverse reactions in Group B suggests that Cordyceps essence could be a suitable alternative for patients who cannot tolerate hydroquinone cream.

Metabolomics explores the relationship between metabolites and physiological changes through qualitative and quantitative analysis, offering a clearer and more direct reflection of an organism's physiology [26]. A total of 404 metabolites were detected, of which 29 showed significant changes after treatment (*p* < 0.05). Differential metabolites revealed three key insights into melasma therapy: First, the reduction of melanogenic precursors including benzoic acid strongly correlated with MASI score improvement, supporting their role in melanin regulation [2]. Second, elevated antioxidant metabolites such as arginine and citrulline paralleled skin lightening effects, consistent with oxidative stress mechanisms in melasma [27]. Third, increased histidine levels were associated with improved skin barrier function, aligning with recent findings on barrier dysfunction in melasma pathogenesis [28]. These findings collectively demonstrated how Cordyceps essence modulated multiple pathways to improve melasma.

Of the 49 metabolic pathways analyzed, 15 showed significant differences after treatment, with the mTOR signaling pathway, β‐alanine metabolism, and arginine biosynthesis pathway being particularly relevant. The mTOR signaling pathway plays a critical role in the growth, proliferation, and differentiation of skin cells. Dysregulation of mTOR signaling is implicated in a wide range of skin conditions, including inflammatory skin diseases and skin cancers [29]. The balance and morphology of the skin rely on the normal function of mTOR signaling [30]. β‐alanine, a non‐amino acid and key component of carnosine, has potential as a topical treatment for skin aging and offers various skin benefits, including antioxidant, anti‐inflammatory, and immune‐regulating properties [31]. Arginine is essentially involved in many catalytic, regulatory, and structural functions. It promotes the proliferation of dermal fibroblasts, has anti‐apoptotic effects, and enhances immune defense function [32]. Additionally, arginine serves as a precursor to creatine, ornithine, and polyamine, which are vital for collagen synthesis, cell proliferation, and overall skin homeostasis [33].

This study suggests that the effectiveness of combined oral TXA with topical Cordyceps essence in treating melasma may be closely related to improvements in endogenous differential metabolites in skin tissue and the regulation of amino acid metabolism. The observed changes in metabolites and metabolic pathways after treatment could also point to potential therapeutic targets, providing a basis for further mechanistic research.

The limitations of this study are as follows: (1) All participants were female, and the sample size was small, which may limit the generalizability of the results. (2) The study relied on MASI scores to evaluate the severity of melasma, a subjective assessment method that may introduce bias. (3) Due to the limited study duration, the effectiveness and safety of the treatments were only evaluated after four treatments. Further follow‐up is necessary to assess long‐term efficacy and recurrence rates.

## 5 Conclusions

Our clinical trial found that oral TXA combined with topical Cordyceps essence and oral TXA combined with topical hydroquinone cream significantly improved melasma in women, with no significant difference in efficacy between the two treatments and similar levels of patient satisfaction. However, the incidence of adverse reactions was lower with the use of topical Cordyceps essence compared to hydroquinone cream. Cordyceps essence appeared to be a promising alternative for patients intolerant to hydroquinone cream. Metabolomic analysis revealed that modulation of melanogenesis‐related metabolites, enhanced antioxidant activity, and improved skin barrier function collectively contributed to the clinical improvement in melasma severity. The effectiveness of oral TXA combined with topical Cordyceps essence in treating melasma may be closely linked to its ability to improve endogenous metabolite levels in the skin and regulate amino acid metabolism.

## Author Contributions

Sihao Shen designed the research and performed the reasearch. Huiyi Yao and Yuan Zhu contributed to data collection. All authors have read and agreed to the published version of the manuscript. Wenzhong Xiang carried out critical revision.

## Ethics Statement

This study was approved by the Medical Ethics Review Committee of the Hangzhou Third People's Hospital and complied with relevant laws and regulations in China and the Helsinki Declaration to protect the rights and interests of subjects (number: 2023KA022).

## Conflicts of Interest

The authors declare no conflicts of interest.

## Data Availability

The data that support the findings of this study are available from the corresponding author upon reasonable request.
